# Sensory Abilities of Horses and Their Importance for Equitation Science

**DOI:** 10.3389/fvets.2020.00633

**Published:** 2020-09-09

**Authors:** Maria Vilain Rørvang, Birte L. Nielsen, Andrew Neil McLean

**Affiliations:** ^1^Swedish University of Agricultural Sciences, Department of Biosystems and Technology, Alnarp, Sweden; ^2^Université Paris-Saclay, INRAE, AgroParisTech, UMR Modélisation Systémique Appliquée aux Ruminants, Paris, France; ^3^Equitation Science International, Tuerong, VIC, Australia

**Keywords:** equitation science, olfaction, auditory, tactile stimuli, visual stimuli, human-animal relationship, welfare

## Abstract

Vision, hearing, olfaction, taste, and touch comprise the sensory modalities of most vertebrates. With these senses, the animal receives information about its environment. How this information is organized, interpreted, and experienced is known as perception. The study of the sensory abilities of animals and their implications for behavior is central not only to ethology but also to animal welfare. Sensory ability, perception, and behavior are closely linked. Horses and humans share the five most common sensory modalities, however, their ranges and capacities differ, so that horses are unlikely to perceive their surroundings in a similar manner to humans. Understanding equine perceptual abilities and their differences is important when horses and human interact, as these abilities are pivotal for the response of the horse to any changes in its surroundings. This review aims to provide an overview of the current knowledge on the sensory abilities of horses. The information is discussed within an evolutionary context and also includes a practical perspective, outlining potential ways to mitigate risks of injuries and enhance positive horse-human interactions. The equine sensory apparatus includes panoramic visual capacities with acuities similar to those of red-green color-blind humans as well as aural abilities that, in some respects exceed human hearing and a highly developed sense of smell, all of which influence how horses react in various situations. Equine sensitivity to touch has been studied surprisingly sparingly despite tactile stimulation being the major interface of horse training. We discuss the potential use of sensory enrichment/positive sensory stimulation to improve the welfare of horses in various situations e.g. using odors, touch or sound to enrich the environment or to appease horses. In addition, equine perception is affected by factors such as breed, individuality, age, and in some cases even color, emphasizing that different horses may need different types of management. Understanding the sensory abilities of horses is central to the emerging discipline of equitation science, which comprises the gamut of horse-human interactions. Therefore, sensory abilities continue to warrant scientific focus, with more research to enable us to understand different horses and their various needs.

## Introduction

**Figure 1:**
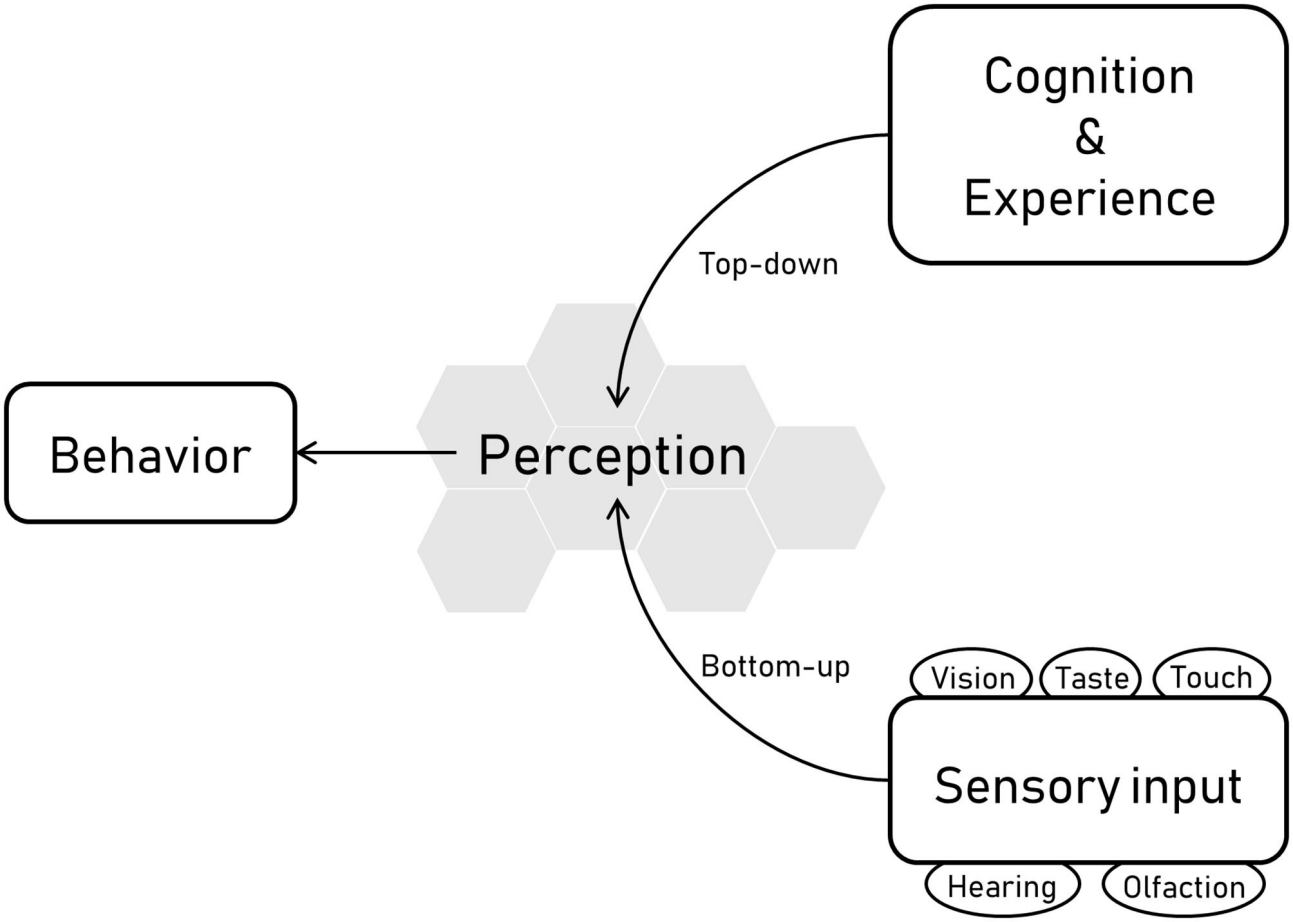
Overview of the links between behavior, perception, and sensory information. The sensory abilities of horses are linked with their perception and therefore their behavior. Sensory receptors related to vision, hearing, olfaction, taste, and touch receive and process information from the surroundings, and this input is organized, interpreted, and consciously experienced, which is what is referred to as perception. Perception comprises both bottom-up and top-down processes, where bottom-up refers to the processing of sensory input into perceptions, and top-down processing refers to perception that arises from cognition i.e. influenced by knowledge and experiences.

The senses of an animal refer to the sensory apparatus by which the animal receives information about its environment. For most vertebrates these comprise vision, hearing, olfaction, taste, and touch, although some species have additional sensory modalities, such as electroreception in some fish species, magnetoreception in some bird species, sonar in cetaceans and some bat species and infra-red capabilities in some reptile species. Sensory receptors are constantly bombarded with information from the surroundings, and how this input is organized, interpreted, and consciously experienced is what is referred to as *perception* (1). Perception functions both as a bottom-up and a top-down process; bottom-up refers to the processing of sensory input into perceptions, whereas top-down processing refers to perception that arises from cognition i.e., influenced by knowledge and experiences (Figure 1). Understanding the sensory abilities of animals and what these abilities mean for the behavior is central not only to the science of ethology but also to the study and assessment of animal welfare, which refers to “*how an animal is coping with the conditions in which it lives”* (2).

The sensory abilities of horses are closely linked with their perception and therefore their behavior (Figure 1). Horses and humans share the five most common sensory modalities, but their range and acuity differ between the two species, so that horses are unlikely to perceive their surroundings in the same way as we do. Although it is often assumed otherwise, equine sensory abilities are different from those of humans, and hence a better understanding of the sensory abilities of horses is fundamental to horse-human interactions and broadly in equitation science, particularly in light of the emerging focus on positive welfare. Equitation science promotes an objective, evidence-based understanding of the welfare of horses in their interactions with humans by applying valid, quantitative scientific methods (3). Despite horses having been described in the past as one of the most perceptive of animals (4), research on equine sensory abilities is limited, and has mainly focused on hearing and vision. Olfaction and tactile sensitivity, on the other hand, has only been studied sparsely. Horses have a well-developed olfactory epithelium (5), suggesting an extensive role of the sense of smell, but only few studies have investigated the olfactory capacity of horses, focusing mainly on its relation to reproduction and social behavior. It is also surprising that despite touch being the main means of communication between the rider and the horse, only seven peer-reviewed published studies can be found on this subject.

The understanding of the sensory abilities of horses is also of growing importance in the use of horses in sport and leisure. Welfare concerns surround various practices in horse sports including hyperflexion of the cervical vertebrae, the use of tight constrictive nosebands (6) and in horse racing the use of the whip has come under increasing scrutiny (7).

This review aims to provide an overview of research on the sensory abilities of horses. Current knowledge will be presented in relation to equitation science and also within an evolutionary perspective in order to understand why these sensory capacities have evolved, and to outline gaps for future research. Perhaps most importantly, this information is put into a practical context outlining potential ways to reduce the risks caused by insufficient knowledge of the sensory perception of equines, which can create dangerous situations for both humans and horses.

## The Equine Sensory Apparatus

### Vision

Vision is the most widely studied sense in horses. Scientific research has mainly focused on color vision (8–12), depth perception and visual acuity [reviewed by Timney and Macuda (13)]. There have been limited studies on interocular transfer (14), and scotopic vision (15). Interestingly, the absence of interocular transfer (i.e., the ability to habituate to a stimulus observed through only one eye) in horses has been anecdotally noted by many horse trainers, however research is scant and conflicting. Further studies into this important area are required because of its relevance for ridden and led horses in terms of assuming that habituation via one eye transfers to the other.

#### Panoramic Field View and Acuity

Life as a large cursorial ungulate living in mainly open habitats such as grasslands, presents unique challenges for survival. In such an environment, predators have the advantage of being able to constantly monitor the position and movements of prey such as horses. Unsurprisingly therefore, horses have evolved sensory abilities that are optimal for predator detection and escape. Equine visual abilities provide the perfect example of such adaptations.

The eye of the horse is among the largest of terrestrial vertebrate species (16, 17). Contrary to the binocular and typically narrowed vision of most predators, the evolution of the horse's visual field has favored a more panoramic field of view with only limited binocular capability. Anatomical studies have shown that the maximum extent of the uniocular field of view in the horse is 228° with a mean around 195° (13). The binocular field of vision, which is 120° in humans, is only 55° to 65° in front of the horse (18), and the overlap is predominantly below the head, extending down ~75° (13). The visual input is therefore narrow and wide conferring a panoramic view, with only a small blind spot at the rear.

In the early studies of equine visual abilities, most authors argued that horses had poor acuity [e.g., (19, 20)] owing to the low density of cones in the retina. Later behavioral acuity studies, together with measurements of ganglion cell density and electrophysiological measures have confirmed these assumptions (13), indicating that horses have poorer acuity than most other terrestrial mammals. Hence at first glance, it seems somewhat surprising that horses are so capable in showjumping and eventing competitions where jumping obstacles indisputably requires substantial visual abilities to gauge both distance and height of obstacles. However, studies of depth perception in horses reveal that horses possess true stereopsis, i.e., the ability to perceive depth and 3-dimensional structure obtained on the basis of visual input from both eyes (21), thus only within the binocular vision field.

In terms of equitation science, the relatively limited binocular vision and visual acuity may help explain the higher frequency of faults at obstacles with successive elements in combination [i.e., closely-spaced consecutive obstacles; (22), and wall obstacles (23)] notwithstanding the effect of human judgment error.

As opposed to the human retina and its central fovea, the equine retina has no central fovea but instead has what is known as a “visual strip” (24). This gives the horse the ability to broadly and most likely equally see the entire horizon, but much less above or below. From an adaptive viewpoint, this horizon-focused vision has obvious benefits for an open grassland prey animal with no aerial predators and little threat from beneath. To bring an object into focus, the horse will usually lift, lower, or tilt its head to make use of the visual strip. Head and neck position are therefore important factors found to affect the visual abilities of horses. In 1999, Harman et al. (24). questioned whether the over-arched neck of the ridden horse in the sport of dressage would inhibit the horse's ability to see what is directly in front of it. The trend in dressage over the last few decades has been for increasing arching of the neck (dorsoventral hyperflexion of the cervical vertebrae), resulting in the nasal planum keeping behind the vertical line (>90°). Research [e.g., (25)] has highlighted the visual deficits that occur when the angle of nasal planum increases beyond the vertical line. Bartoš et al. (26) challenged this assumption and found that 16 riding school horses were not visually impaired when ridden with a vertical nasal planum (~90°) because a horse is able to rotate its eyeball, enabling a horizontal eye position and hence a horizontal field of vision. What the authors did not investigate however, were head/neck positions >90° also called “behind the bit.” More recent findings suggest that the rotation of the eyeball can compensate for some head and neck rotation, but not the most extreme hyperflexed positions. In these cases, the pupil (and hence the field of vision) is no longer parallel with the ground (27). In contrast to the more fixed position of dressage horses, riders in showjumping and eventing typically allow their horses sufficient rein so that they have the freedom to choose their own head carriage appropriate for clearing the obstacle. This is particularly important just before and during the jumping effort as it enables the horse to achieve optimal athleticism and balance when negotiating an obstacle.

From an adaptive viewpoint, stereopsis would facilitate sure-footedness for a swift large cursorial ungulate where predators have the advantage of surprise. Stereopsis also enables the horse to pinpoint and evaluate the potential threat. This may also explain the alert behavior where the horse stands vigilant with an elevated neck, and with head and ears oriented toward the stimulus [i.e., a potential threat; (28)].

#### Night Vision and Color Vision

Horse pupils can dilate greatly to capture sparse photons at night, and the retina is generally rod dominated (29). In addition, the reflecting tapetum lucidum (Latin for “bright tapestry”) in the back of the horse's eye, gives the non-absorbed photons a greater likelihood of capture by photoreceptors, thereby enhancing sensitivity further (30). All these features result in good scotopic vision, i.e., ability to see under low light conditions. This is another adaptive trait that has been favored by natural selection given the nocturnal hunting proclivities of canine and feline predators. Equine scotopic abilities were first deduced from behavioral observations of free-ranging horses that maintained their grazing and interactions with conspecifics at nighttime (31, 32). Later, studies noted that horses see details better on overcast days as opposed to bright sunny days (33). The horse has a higher proportion of retinal rod cells than humans, resulting in superior night vision. One of the more recent studies indicates that horses and humans have similar thresholds, being able to discriminate colors in light intensities comparable to that of moonlight (34), nevertheless horses are still able to see objects at lower light intensities than humans. More recently, this suggestion was tested by Hanggi and Ingersoll's (15) research showing that horses can solve two-dimensional discrimination tasks in nearly complete darkness. Horses also possess good visual capacity under both natural and artificial light conditions (35).

Grzimek (12) was among the first to show that horses have color vision, and several studies have since confirmed the ability of horses to see some colors [e.g., (8–11, 36–38)]. Equine color vision is dichromatic (i.e., color vision that is deficient in one of the three cone pigments e.g., “red-green blindness”), resembling that of red-green color-blind humans (9). This is an important aspect to consider in eventing and showjumping when choosing the colors of obstacles, as these may not be as obvious to the horse as they are to the rider. Several studies have also shown that obstacle color affects how likely the horse is to detect, and hence how successful the horse jumps the obstacle. Stachurska et al. (39) found that it can be difficult for horses to jump obstacles which are all light or all dark, as uniform light may cause an optical illusion, which overestimates the size of the obstacle, and uniform dark may make the horse disregard the obstacle altogether. Furthermore, these authors suggested that the color of the surroundings plays a crucial role. The horses they tested seemed to detect blue-colored obstacles easier than green, which they conclude is due to the coloring of the (grassed) jumping arena. Paul and Stevens (40) tested racehorses on obstacles colored orange, fluorescent yellow, bright blue, or white, and found that the obstacle color influenced both the angle of the jump and the distances jumped. Like Stachurska et al. (23) they found that white resulted in the largest takeoff distance, and bright blue produced a larger angle of takeoff, whereas jumps over fluorescent yellow fences had shorter landing distances compared to orange. Altogether, these studies highlight that coloring of obstacles is of high importance, especially when evaluating the level of difficulty of the tracks. Although obstacles might seem easy/hard to the human eye, the coloring alone might result in an easier/harder challenge than anticipated. Such considerations are important in eventing, stadium (show) jumping and other jumping sports including steeplechasing and hurdling.

A research field that has received increasing attention in recent years is visual laterality in horses. These studies suggest a correlation between emotion and visual laterality when horses observe inanimate objects. Austin and Rogers (41) found that horses were more reactive to a fear-eliciting stimulus when presented on the left of the horse. De Boyer Des Roches et al. (42) later showed that horses prefer the left eye for viewing objects that could have both positive and negative associations, and Farmer et al. (43) added that horses prefer the left eye when observing humans or the surrounding environment. These results can help explain the widespread observation that horses often have a preferred side (i.e., motor laterality) on which they are easier to handle [e.g., (44, 45)].

### Hearing

Horses show visible reactions to sounds, with one or both ears typically moving toward the direction of the sound source. The hearing ability of horses was first studied in the 1980's by Heffner and Heffner (46–48) and surprisingly little research has been done on horse hearing since. They mapped the range of frequencies horses can detect and demonstrated that while larger animals tend to be adept at hearing lower frequencies, horses are an exception. The lowest frequency detectable by horses is 50 Hz, which is higher than the lowest human detection threshold of 20 Hz. Conversely, equine hearing exceeds the highest frequencies that can be heard by humans (33 kHz compared to 20 kHz for humans), indicating that there will be situations where a horse can detect sounds that humans are unable to hear, and vice versa. Furthermore, the funnel-shape of the equine ear provides an acoustic pressure gain of 10 to 20 dB (49) improving the acuity of equine hearing. From the viewpoint of horse-human interactions, it is important to consider that the higher frequency hearing abilities of horses compared to humans may explain some of the unwelcome and otherwise inexplicable behaviors that are regarded as problem behaviors.

As a generality, there is an inverse relationship between the mass of a mammal and its hearing frequency threshold (50). The horse represents an outlier in this regard, as a large mammal with limited low frequency hearing but good acuity in the higher frequency ranges. High frequency hearing is undoubtedly adaptive in horses and is likely to provide the horse with important information regarding, among other things, the stealthy advance of predators.

Horses have been found to demonstrate auditory laterality, i.e., by turning one ear more than the other toward the source, when calls from group members, neighbors and strangers were played. A clear left hemispheric preference (i.e., the horse turns its right ear more toward to source) was found for familiar neighbor calls, whereas there was no preference for group member or strangers calls (51). Horses also appear to possess a cross-modal recognition of known individuals. This means that when presented with a visual representation of a known individual, combined with a playback call from another conspecific (i.e., mis-matching), horses respond to the call more quickly and look significantly longer in the direction of the call, than if the visual and auditory cues match (52). This cross-modal recognition was also found to occur when horses were presented with familiar humans. Horses looked quicker and for longer at humans when the auditory cues were mis-matching. This suggests that the equine brain is able to integrate multisensory identity cues from a familiar human into a person representation. This would allow the horse, when deprived of one of its senses, to maintain recognition (53). In practical terms, this means that the horse is able to recognize a familiar person based on vocal cues (e.g., voice), even when unable to see this person, and the same appears to be the case for conspecifics. If horses form individual representation of other horses, this might help explain why some horses react strongly to separation from conspecifics. Although the horse is physically removed, call backs from conspecifics could still affect the horse, and depending on the social relation between the two, cause the horse to behave more aroused or anxious. What remains unknown, however, is the role of olfaction in these studies. As noted by Lampe and Andre (2012), olfaction may act together with a visual cue (i.e., when horses were physically presented with the human), and it would thus be beneficial to design a study that separates the two types of sensory input.

#### Aural Impairment

Old age is known to affect hearing ability in many animals, including humans. In horses, only one study has investigated hearing ability as a function of age, finding that older horses (15–18 years old) showed fewer behavioral reactions to sounds than younger horses [aged 5–9 years; (54)]. Since then, no published studies have investigated age and hearing impairment in horses although several studies have emphasized the importance of hearing [e.g., (55)]. It has been suggested that as deafness progresses, the horse can compensate by enhancing other senses such as vision and by learning daily routines to still behave as per usual (56). Detection of partial or complete hearing loss in horses can be difficult, but it is nevertheless important for people working with horses to be aware that hearing ability can weaken with age. Horses are commonly trained to react to voice commands from the rider/trainer and such commands will become less detectable as the horse ages. Likewise, horses communicate with each other by means of vocalization e.g., during mating and whilst rearing their young, and these are predominantly low frequency sounds (57). Depending on the type of deafness (high or low frequency) horses may show no signs when ridden (high frequency sounds), but still be constrained in their social communication, or vice versa (55).

**Figure 2:**
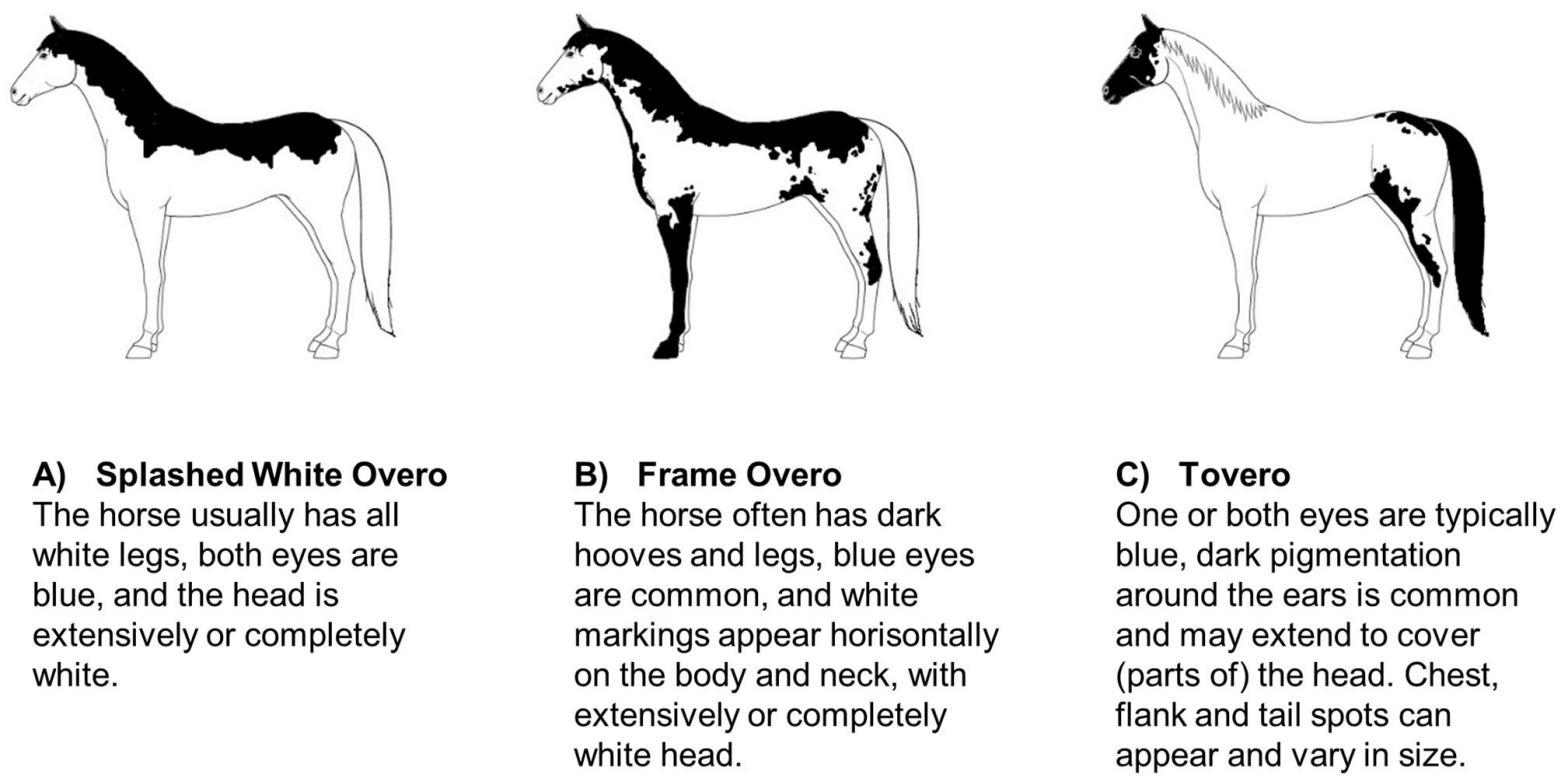
Schematic examples of American Paint horse coat patterns found to be linked to deafness (58): **(A)** Splashed White Overo, **(B)** Frame Overo, and **(C)** Tovero. Coat pattern descriptions are adapted from the official breed descriptions by American Paint Horse Association (59).

Specific coat color patterns have been found to be associated with an increased risk of deafness in some horse breeds. Magdesian et al. (58) investigated 47 American paint horses and pintos, and found that particularly the paint horses with a splashed white or frame overo coat color pattern, a blend of these patterns, or with a tovero pattern had a higher risk of being deaf (Figure 2). Horses with extensive head and limb markings and those with blue eyes appeared to be at particular risk. Whether or not this is specific to the color patterns in general (within all breeds) or to these color patterns within the two breeds investigated is unclear. As these color patterns also occur in other breeds it could be investigated if the propensity for reduced hearing is a more general genetic association across breeds.

#### The Impact of Sound

Noise is over-loud or disturbing sound (60), and it is well-known that loud noises can cause stress responses [physiological and/or behavioral changes to cope with changes in the external environment; (61)] in farm animals (62). Continuous noise can have a negative impact on animal health (63). It has been shown in several studies that noise is a stressor for both pigs [e.g., (64, 65)] and cattle [e.g., (66, 67)]. The potential aversive effects of noises emanating from windfarms are contentious and have been the subject of legal cases throughout the Western world. One of the authors of this article (AM) has acted as an expert witness in such cases in Australia, New Zealand and Ireland, where proprietors of horse facilities adjacent to wind farms attest that the sound (as well as the flicker of moving turbine blades) is aversive to horses and has safety ramifications for horse riders and handlers. Research on the impacts of wind tower-related stimuli however is lacking. Notwithstanding, in a very limited (and not peer-reviewed) survey, the British Horse Society reported that more than 20 percent of riders in their UK sample had experienced an adverse reaction from their horses to wind turbines (68). However, Burton et al. (69) reported that the sound level adjacent to a turbine is about 55 dB and much of the sound spectrum is in the infrasound range (low Hz not detectable for horses). These authors point out that by contrast, a car traveling at highway speed generates about 80 dB. In their survey, the British Horse Society (68) report that the susceptibility of horses to react to wind towers does not seem to be related to the temperament of the horse.

In many horse barns and riding stables, it is common for a radio or other music devices to be playing during the time when people are active. The effect of such music has not been widely studied in horses, and it is therefore unknown if these sounds are perceived as attractive or aversive by the horse. Classical or slow instrumental music have been found to increase milk yield in dairy cows (70) and Country music can facilitate dairy cows' entry into the milking apparatus (71). For horses, only few studies have been carried out. In everyday horse management situations, the effects of music have only been studied by Neveux et al. (72). They found that classical music reduced the intensity of stress responses of horses subjected to either a short transportation or a farrier treatment, suggesting that background music can have practical implications. One study investigated the potentially calming effects of music on ponies, but found no effects of either classical, jazz, country or rock music (73). Stachurska et al. (39) have shown that instrumental guitar music can have a positive influence on Arabian racehorses when played for 5 h per day for a period of between 1 and 3 months, after which the positive effect diminished. The same type of music was tested in a study that showed that the positive effects of playing the music was greater when played for 3 h per day than for 1 h per day (74) confirming the positive effects of instrumental guitar music. Collectively, however, these studies only compared music against silence (in this case meaning no music, which is not nessesarily silence), and hence the treatments were a more general “sound” vs. “no sound” comparison, with the former potentially masking sudden noises from e.g., machinery or slamming of barn doors. Such noises have previously been found to be stressful in other species [in cattle e.g,. (75), and pigs e.g., (76)], and it would thus be beneficial to include a larger variety of sounds in future studies with horses. This could reveal if other sounds than music have a calming effect, and also explore if horses are aversive to sounds that other species find aversive. It would also be worth investigating the effects of classical music in other potentially stressful situations to gauge the magnitude and duration of the positive effects e.g., during longer transportations. This is especially important because one of the benefits of using music as a calming tool is that it can be applied without any humans present.

Although the effects of sound and music on horses are understudied, the anecdotal assumption that horses can spontaneously move to a musical beat is widespread among horse riders and trainers (personal communication), although scientific evidence of this ability is sparse, if not absent. From an evolutionary perspective it would seem an unlikely phenomenon that would entail the recruitment of higher mental processes than those so far found to be possessed by horses. Bregman et al. (77) investigated horses moving to music and noted the footfall and the beats of the music to analyze if horses possessed the ability of synchronizing their tempo to a musical beat. The preliminary results (based on one horse) suggest that a horse may be able to spontaneously follow a rhythm, but more studies with larger sample sizes than in Bregman et al. (77) are needed to refine the method and confirm the findings.

Insect, snake and rodent traps using ultrasound are becoming more and more common in households and in stable buildings, replacing the use of poison. These devices usually emit ultrasound at frequencies above 32 kHz [e.g., Rodent Repeller^TM^, (78)] to ward off pests, but some (mostly rodent repellant devices) use frequencies as low as 18 kHz [e.g., Ultrasonic Electronic High Power Pest Repeller, (79)], which is detectable by horses. It remains unknown to what extent horses detect and perceive noises from these pest repellants, especially when the frequency used is within the equine hearing range of 50 to 33 kHz. This should be investigated in order to ensure no detrimental welfare effects for horses arise from the use of such devices.

### Olfaction

**Figure 3:**
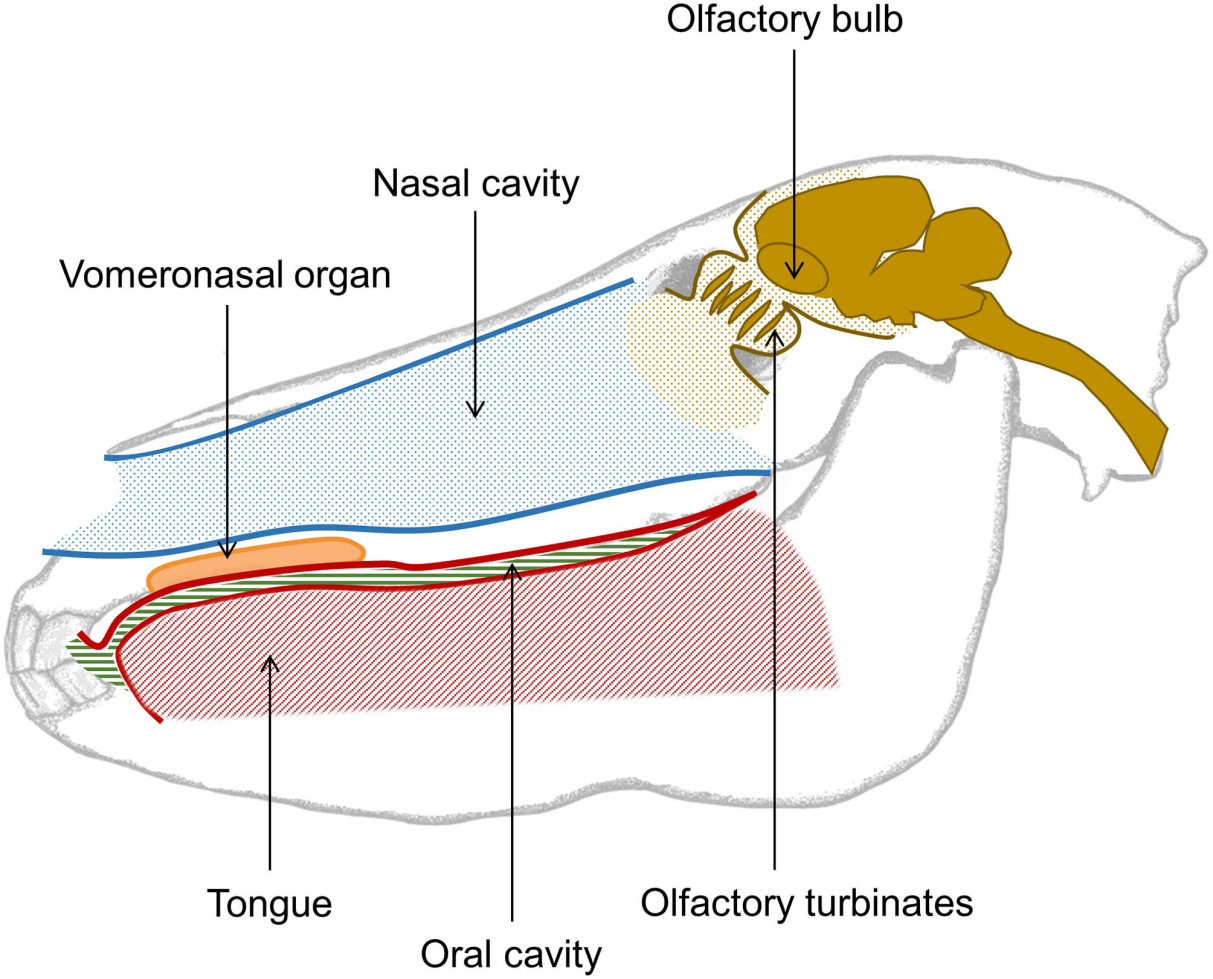
Simple overview of the nasal and oral cavity of the horse. The nasal cavity is opened while breathing and closed while the horse swallows. While breathing, the oral cavity is sealed from the esophagus, hence the tongue takes up most of the space in the mouth. The vomeronasal organ of the horse is situated in the upper jaw, just between the palate and the nasal cavity, and opens behind the front teeth. This organ is used to detect poorly volatile organic compounds and triggers the flehmen response. The olfactory turbinates hold the olfactory epithelium with sensory neurons projecting to the olfactory bulbs localized in the front of the horse's brain. Altogether, this forms the first part of the olfactory system.

Like other mammals, the olfactory organ of the horse consists of an olfactory epithelium lining the inside of the upper nasal cavity and connecting via olfactory neurons held in the turbinates to olfactory bulbs in the horse's brain. It has recently been determined that in horses the tip of the olfactory bulb aligns with the center of the forehead hair whorl (80). Horses also have a well-developed vomeronasal organ (Figure 3), which is receptive to non-volatile and poorly volatile molecules, often found in body secretions (5, 81). When a horse gets into contact with a substance of interest, the associated molecules activates the vomeronasal organ which triggers a flehmen response, i.e., where the horse curls its upper lip back and inhales, often with closed nostrils. The adaptive advantage of the flehmen response is that it enables the horse to analyse poorly volatile compounds with far greater accuracy. During the flehmen response the nostrils are closed thereby reducing the escape of air, and increasing the air pressure within the nasal cavity. This allows molecules from the compounds to be detected by the vomerosal organ.

Such a highly developed olfactory apparatus indicates that information from odors is important to horses, but despite being a central sensory modality, research on olfaction is relatively scarce for this species. Only a handful of studies have examined the role of olfaction in horses, and these have mainly focused on reproduction and social recognition. Marinier et al. (82) found that stallions did not differ in their response to the odor of urine and vaginal secretions of a mare in estrus as compared to when that same mare was not in heat. Later, Briant et al. (83) and Jezierski et al. (84) supported those findings by showing that stallions could not differentiate feces of mares in estrus from those in diestrus. We know that odorant differences exist between these equine feces types, as male rats are able to distinguish between them by smell alone (85). From an evolutionary perspective, the ability to use odors for estrus detection may have been lost (or never existed) in horses, as stallions either lead a predominantly female harem or belong to an all-male group of younger horses. When surrounded by mares, odors may be unnecessary for estrus detection as only mares in estrus will allow a stallion to mount her. Stallions thus rely on the mare's behavioral responses when determining whether or not she is ready for mating.

Odors are, however, used to some extent by horses for social recognition, as they are able to distinguish between different individuals by their smell. Jezierski et al. (84) tested stallions' responses to feces of both sexes and found that mares' feces were sniffed for longer. The stallions also expressed more flehmen behavior when sniffing mare feces than when sniffing stallion feces, and urination on feces happened exclusively when it originated from mares. In contrast, Krueger and Flauger (86) investigated odor discrimination and found that although horses were able to distinguish their own feces from that of conspecifics, they were not able to differentiate between the feces of unknown vs. familiar horses, nor were they able to distinguish mare feces from that of geldings. Studies of feral or free ranging horses have previously described how these animals recognize each other on the basis of body odors (87, 88), as well as odors from urine and feces (86, 87). Moreover, Krueger and Flauger (86) showed that horses exhibited more interest in the feces of horses from whom they received the highest amount of aggressive behaviors. The authors concluded that horses of both sexes can distinguish individual competitors among their group mates by the smell of their feces, in accordance with previous findings (89, 90).

Volatile organic compound profiles from hair samples have been found to differ among horse breeds, and these odor profiles are different in cohorts of related compared to non-related horses (91). The odor profiles indicate a degree of kinship (92, 93), suggesting that each horse has its own odor profile with a certain degree of similarity among related individuals. This ability to recognize conspecifics based on odor can be used by the horse to guide its response with other horses in the group based on previous experiences, so that odor profiles become an aid in determining the potential outcome of a given interaction (91). Individual olfactory recognition can therefore be considered an evolutionary beneficial trait, which still exists in domestic horses. Odors from different horses should be taken into consideration during their handling, as they will leave a scent trace on the human handler. A person training many horses a day will end up with many different odor traces on their clothes, hands and on equipment, and these odors may affect horses handled subsequently, such as if an early-handled horse is a known aggressor.

#### Familiar and Calming Odors

Hothersall et al. (87) were the first to develop a Habituation-Dishabituation test (termed *Habituation-Discrimination* test in the original paper) for horses and found that mares could distinguish between urine samples from other pregnant mares and from geldings. Interestingly, this testing paradigm has not been subsequently used to test odor discrimination in horses. The olfactory capacity of horses could be exploited in different situations if more knowledge about odor detection and preferences were known. Attractive smells could potentially draw horses to certain places/locations scented with these odors, limiting the need to manually move the horses e.g., during regrouping where the presence of a human handler could pose a safety risk. Moreover, conditioning horses to associate a certain odor with a pleasant experience could take the use of odors a step further. Odorant conditioning has barely been explored in livestock, but rats can learn to associate an odor with positive human tactile stimulation (94). Such positive odor conditioning has the potential to be used as an alternative to food rewards or as a calming addition in otherwise stressful situations. Horses could be conditioned to associate a specific odor with positive stimuli such as grooming, feeding or social comfort, and the same odor could potentially be applied during stressful or fear-eliciting situations such as trailer loading, regrouping, and social isolation.

One such allegedly calming aid is pheromone spray or gel. These products claim to have a calming effect on horses, but research has yielded conflicting results. Falewee et al. (95) tested one such commercial pheromone (0.1% solution as a spray) in a controlled study of 40 horses and found significantly lower heart rates and less fear-related behavior in the horses treated with the pheromone. Collyer and Wilson (96) later tested a pheromone gel on horses thought to be experiencing separation anxiety when they (four tightly bonded pairs) were removed from each other and found no significant effect, except for a tendency for the product to dampen extreme anxiety. Berger et al. (97) tested the pheromone spray during abrupt weaning of foals (*n* = 14) and found no significant effects of the pheromone treatment on behavioral measures nor cortisol concentration. More efficacy testing of such odorant products is needed, and this should include effects of age and breed of horses and means of application of the product. The masking effect of these odors should also be taken into account in these studies, as the mere presence of any unknown smell may give rise to similar behavioral changes. Pheromones are usually thought of as eliciting an innate and biologically meaningful response; however the behavioral response can also be learned (93). As suggested earlier, exposure to an odorant compound in combination with calming stimuli may be needed for the horse to form the association and elicit the calming effect (98).

Another, mostly unexplored area is odor imprinting in young ungulates. Odor imprinting has, to our knowledge, not been studied in horses and only sparsely in other mammalian species. A black-tailed deer fawn reared and bottle-fed by (or in the presence of) a surrogate deer with pronghorn odor later showed preferences for pronghorns over its own species (99), demonstrating the lasting role of odors for the formation of preferences. Imprinting the odors of future human handlers on foals may induce long-lasting preferences, which could potentially calm young horses. This type of imprinting could be further developed if the foal is subsequently conditioned to associate the human odor with a positive stimulus.

#### Aversive Odors

In mammals, the most well-known non-learned (i.e., innate) response to an odor is the avoidance of, or flight from, the smell of a predator (100). Such innate responses are adaptive, and studies indicate that the ability is even preserved in species living where no predators have been present for centuries (101). Horses have also shown vigilance behavior when exposed to an unknown odor [eucalyptus oil; (102)], and to a predator odor [wolf urine; (103)]. Pairing a predator odor with a loud noise elicits significantly higher heart rates in horses than when only exposed to one of the stimuli (103), suggesting that the mere presence of a predator odor can increase the response to fear-eliciting situations. This would indicate that when in an environment where predators roam, the horse may be more reactive than usual, and detection of predator odors may be one of the reasons why horses react unpredictably or more abruptly in some situations. Riding in or close to environments where the likelihood of encountering canid or felid predator odors is higher may pose a safety risk to horse and rider.

It is commonly speculated that humans, when scared or stressed, secrete odorous compounds associated with fear, which can affect the horse (5). Several studies have shown an increase in heart rate of horses when either handled or ridden by a nervous person (104, 105) and similar increases have been seen in horses when stroked by a negatively thinking person, which in this study were male subjects with a negative attitude toward companion animals in general (106). Contrary to these findings, and perhaps surprising to many, Merkies et al. (107) found that horses react more calmly (measured as both relaxed behavior and lowered heart rate) when accompanied by a stationary nervous or physically stressed person than a calm person. Although these are preliminary results, the authors question the common saying that horses will be scared if the person is scared. Even when a person is stationary, subtle movements and body language of the human is likely to affect horse/human interactions, and this may have influenced the results. Horses express more relaxed behavior in the company of humans who express a positive attitude toward horses (108). Some of the conflicting experimental data may be explained by breed differences, for example Merkies et al. (107) used draft horses whereas other studies used warmblood, riding/sports horses. It is nevertheless interesting that none of the studies above considered the potential effect of human odors on horse behavior. In a recent study, Sabiniewicz et al. (109) presented horses (*n* = 21) with samples of human body odor collected under either fearful or happy conditions. The horses showed different behavioral responses to the two types of human odors, and the authors suggest that domestication of horses may have favored the ability to recognize heterospecific emotions.

The male hormone testosterone, or its derivatives such as androsterone, are known to have specific pheromonal effects in various species: It can accelerate estrus in multiparous cows [reviewed in (110)], stimulate lordosis in pigs [e.g., (111)] and reduce anxiety behavior in male rats [e.g., (112)]. It would be interesting to explore effects of testosterone, as well as other sex-related compounds, on horses in order to ascertain if horses react differently to men and women. It is likely that humans wearing artificial odors, arising from shampoos, soaps and deodorants, will hide the natural human odors. This could have both positive and negative consequences as it can mask potential human odors connected with fear and stress but also limit imprinting and other familiarity benefits. Further studies of the type carried out by Sabiniewicz et al. (109) within a controlled setting for human body odors as well as handler movements are needed to disentangle these different effects.

### Taste

Humans are able to associate some odors with a certain taste, and vice versa, and refer to the combined effect of smell and taste as flavor. Unlike humans, horses only breathe through their nostrils (Figure 3), and oral breathing only occurs if the horse is physically prevented from nasal breathing (113). The tasting organ of horses is ontogenetically linked to the olfactory epithelium, but it is not known if horses are able to associate odor and taste and form a concept of flavor like humans. However, even though horses do not smell retro-nasally while masticating, odors are likely to escape the mouth, allowing the horse to smell what it eats, and this may connect taste and smell. Indeed, horses are capable of detecting four of the five taste components i.e., sweet, sour, salty, and bitter, whereas detection of umami (a kind of savory taste) in equines is as yet unknown. Like many other ruminant species (e.g., sheep), individual horses are quite variable in their responses to a particular taste (114). The greatest variation in individual taste preferences (in this case pellets) was found in purebred Arabian horses (115), indicating that breed differences are present. Generally, flavor affects diet acceptance and consumption time of horses (116), but when comparing taste, odor, and nutrient contents, the latter has been shown to be the main driver for horse diet choices (117). Flavor can also be used to condition a horse's food aversions such as when lithium chloride is used to avert horses from grazing locoweed (118), and conditioned taste aversion can be a useful management tool when horses are grazing rangelands contaminated with poisonous plant species. The method needs to be applied correctly, as most animals, including equines, learn the aversion only if the feed make them sick shortly after consumption (119).

### Tactile Perception

The skin is the largest organ in horses as well as humans, and the body surface of the horse is thus the largest of the sensory organs. From the evolutionary standpoint, as a prey species it is unsurprising that the horse is a tactile-sensitive animal and has excellent operant conditioning abilities, particularly in negative reinforcement (learning via the removal of an aversive stimulus). Such tactile sensitivity may have served the adaptive purpose of resisting and disabling entrapment by predators, however it also serendipitously foreshadowed the horse becoming the most popular and ubiquitous ridden animal. Tactile stimulation of the surface of the skin is the main interface of communication between a horse and a rider, and also between a horse and human handler.

The sensitivity of the skin is thought to vary across the body of the horse as the distribution of sensory nerve receptors vary, with areas such as the muzzle, neck, withers, coronets, shoulders, lower flank and rear of the pastern typically being most sensitive (120). The skin is sensitive to both thermal and mechanical stimulation. Horses have much thicker epidermis on the trunk than smaller species [e.g., twice as thick as that of cats and rodents (121), which shields them from thermal stimuli]. Mapping of the horse's body show responses to thermal stimulation of the skin when slow heating rates are used, indicating that the responses are mediated mainly by C fibers, (as opposed to Aδ fibers that mediate fast heating) [reviewed in (122)]. This may be why many horses do not react immediately to procedures such as hot iron branding or freeze branding, as the nociceptive threshold is not reached by the fast peak in increasing/decreasing temperature, whereas nociceptive responses are often seen after the exposure. Testing the nociceptive thresholds in horses using heat/cold stimulation is therefore complicated as burns are not easily avoidable (122). Nociceptive thresholds are therefore often tested via mechanical stimulation e.g., using a pressure algometer (123, 124). This method has proved to be a sensitive method for detecting musculoskeletal back pain although it can be confounded by avoidance learning by the horse (125).

In the facial area where the epidermis is thinner, tactile sensitivity is particularly high around the eyes, nostrils and mouth. Like many mammals, horses have vibrissae [also called whiskers; (57)] around the muzzle, as well as around the eyes, but only few studies have looked into their role. It is known however that vibrissae have different characteristics to hair follicles not only in that they are thicker, but also that they are not molted and have greater enervation. For this reason, they are considered as sense organs and removing or thinning them for esthetic purposes has negative welfare implications. Another tactile concern for the area around the nose and mouth of the horse, is the use of restrictive nosebands. Recent studies have shown that nosebands in several equestrian sports are excessively tightened (6) to the extent that natural oral behavior is inhibited, stress can be induced (126), and tissue damage may occur (127). Interestingly, while nosebands are believed to lead to lighter rein tension and to improve control, the modern trend in dressage, eventing and jumping of increased noseband tightness has welfare implications and warrants further investigation.

It is anecdotally believed among horse people that certain coat colors are associated with greater skin sensitivity, e.g., chestnut colored horses (also known as sorrel) are believed to be more sensitive and reactive. While there has been no research in this area in horses, research in mice shows that indeed red coat color is associated with greater pain sensitivity (128) and it would be interesting and important to explore this further in horses. Importantly, it is universally believed in horse-riding sports and traditions that the posture and position of the rider has a profound effect on the horse's ridden responses and behavior. The role of learning theory is well-documented with regard to the controlling stimuli from the rider's reins (via the “bit”), legs, whip and spur. However, there are currently no data clarifying the precise effects of rider posture, although there is considerable literature on various specific characteristics of riders (such as musculature recruitment, balance and forces) whilst riding in the various equine gaits [e.g., (129–133)]. Given the sensitivity of the horse, this represents another important area to pursue in future equitation science.

#### Positive Tactile Stimulation

Grooming or mutual grooming (either between two horses or between a human and a horse), is commonly considered a positive behavior. Non-human mutual grooming has been used as a measure of social bonding in various studies (134, 135). Feh and de Mazières (136) identified an area around the withers of the horse, where grooming caused a drop in the heart rate of the animal, implying a calming effect. On the other hand, Feh and de Mazières (136) also noted that this drop was not present when grooming was done on the shoulders, an area where mutual grooming is commonly directed (137). Normando et al. (138) confirmed the calming effect of grooming on the wither area of saddled horses, but also found a lowering of the heart rate when saddled horses were groomed on the shoulder and hip area. More recently, Thorbergson et al. (139) found that horses under saddle (only standing not ridden) expressed more relaxed behavior when groomed, but as these horses also expressed the same level of agitated behavior as horses not groomed, the results remain unclear. The publications cited here clearly highlight a need for further studies. There may be an unexploited potential for using tactile stimuli much more than is currently done, e.g., as a positive reward. Christensen (140) noted that foals can be easily distracted by scratching their tail region, to which the foals react by lifting the tail and leaning toward to the handler. If tactile stimulation is applied in the correct way i.e., mimicking mutual grooming or scratching at a preferred/itchy spot on the horse's body, it is categorized as primary reinforcement because of its innate reinforcing qualities. Moreover, when applied correctly, such grooming can be used as a positive reinforcer (141, 142) allowing the human handler to avoid or reduce the use of food items as a reward. This is particularly relevant because feeding motivation declines over time, differs between individuals (143), is withheld at certain times during training and can have deleterious effects (144). It should be noted however, that the reinforcing value of tactile stimulation may also show individual, motivational and temporal variation. Another aspect to take into consideration is the recent finding that horses possess sensory laterality in terms of tactile stimulation during affiliative interactions. In affiliative situations, defined as mutual grooming, swishing flies for one another, and standing in close proximity (<2 m away) while grazing or resting, horses showed a significant left eye laterality (145). This finding may help to clarify if the horse perceives a given tactile stimulation as positive. Lastly, although tactile signals have been used for millennia as the major means of communication with horses, given the acute aural and visual capabilities, it may be time to change our ways of communicating with horses. Research into the relative salience of these modalities would be not only interesting but also ultimately useful in determining efficiency and optimal welfare in horse-human interactions.

Another potentially positive tactile stimulation is massage. Massage therapy as a relaxing aid in humans is well-researched and established, and is also used as a method to relieve stress [e.g., (146)]. Massaging horses is not a new trend, and its effects may be embodied in certain forms of horse grooming. McBride et al. (147) showed in a preliminary study that in low to medium stressful situations (defined by the authors as veterinary visits or isolation), massage may be a beneficial tool to alleviate stress in horses. A study has shown that massages every 3 weeks can have a relaxing effect on racehorses, but that daily massages had a stronger positive impact than the less frequent massages or playing relaxing music (74). Research on other animal species have shown that gentle stroking of cows on the head and neck region is perceived as positive by the cow and can enhance their well-being (148). Studies into the neuroendocrine and physiological pathways related to pain and stress further indicate that oxytocin, which is believed to have health promoting effects [e.g., human research: (149–151)], is elevated in the circulation following touch, light pressure and massage-like stroking [sheep: (152); rats: (153, 154)]. In horses, this field of research is new and hence knowledge is limited. Watson and McDonnell (155) performed wither scratching, and face and eye rubbing during confinement in an experimental setting while exposed to a 3-min aversive auditory stimulus (sound of a sheep shearing device). Although no significant effects were found on heart rate, all calming interventions were effective in reducing avoidance and stress responses. Positive tactile stimulation therefore has potential not only as a reward, but also as a stress relieving aid in many equine disciplines, as well as in equine therapy and as a research tool. Research into this area could elucidate its best use, by testing different situations, breeds and protocols of equine massage. Furthermore, in the dog-human relationship, the role of attachment theory and the consequent welfare and safety benefits of secure attachment have been well-documented (156–158). Similarly, as a social species the need for research into the horse-human relationship is urgent (159). Such research may explain the intriguing phenomenon of the horse-human bond and whether or not the horse-human relationship exhibits any of the four features of attachment theory such as: a secure base (from which to explore); a safe haven (to return to when stressed); proximity (the horse stays nearby) and separation distress (stress when separated). Such research may improve equine welfare and the effectiveness of horse-human interactions.

#### Unpleasant Tactile Stimulation

Just as pleasant tactile stimuli can be used in a positive way, some tactile stimuli are perceived as unpleasant. For example, Mayes and Duncan (32) found that feeding patterns in semi-feral horses were influenced by the presence of biting flies. It is thought-provoking that, as horse trainers, we expect the horse to readily habituate to the pressure of the girth, whilst at the same time remain sensitive to pressure from the rider's legs at approximately the same location. The reaction of horses when trying to avoid unpleasant tactile stimulation (e.g., when detecting a fly landing), is tail swishing, skin rippling, ear flicking, foot stomping, head shaking, and biting directed at the particular spot (5). These behaviors are also typically the behaviors used as indicators of possible conflict between the rider and the horse [e.g., (160)]. A significant contemporary issue in equitation science surrounds the use of the whip in horse racing and in particular because the pain [pain can be defined as “an unpleasant sensory and emotional experience associated with, or resembling that associated with, actual or potential tissue damage” (161)] is frequently inescapable [that is, it cannot be switched off by offering a response such as going faster; (7, 162)]. Generally, correct use of aids such as the wip but also spurs and leg aids are important to safeguard the welfare of the horse by offering an opportunity to escape the pain e.g., by moving in the wanted direction.

Another commonly used aid which is based on unpleasant tactile stimulation, is the use of electric shock in form of non-lethal electric fences to control horses on pasture. Horses (and animals in general) quickly learn to avoid the negative experience of contact with an electric fence (163), which makes it a very common and usually accepted mean of controlling horses. In addition, non-lethal electric shock devices have also been used to move animals from one point to another and have even been reported in the popular press in horse racing where such devices known as “buzzers” or “jiggers” have been used to enforce horses to accelerate. Electrically induced pain is painful in an unusual way. Whereas, noxious stimuli are normally transduced into a pattern of action via nociceptors and mechanoreceptors, electrically inducted pain bypasses the transducer mechanisms and induces a reactive pain response (164). The highly unpleasant sensation of pain results from complex interactive mechanisms along the entire neuroaxis, from the periphery to the brain (164) and animals respond in multiple ways including reactive spinal reflexes and affective, avoidance and escape responses (165).

In order to immobilize horses e.g., in order to give an injection, twitching of the skin (pinching and twisting the skin), ear twitching and nose twitching are still deployed. Nose twitching involves pinching the horse's upper lip using a loop rope, chain or other mechanical devices, and is the most commonly used method. As the facial area of the horse, especially around the mouth, is highly sensitive (57), it is worth investigating the underlying neurophysiological processes that underpin the efficacy of twitching. Using the twitch, the person takes advantage of this area being rich in three types of nerve endings detecting pressure, touch and pain. Endorphins are probably involved in the effectiveness of the twitch (166), but regardless of the pathways involved, the twitch likely works because it is painful (167) or because the animal is flooded with sensory information overshadowing all other stimuli that are presented to the horse.

While twitching immobilizes the horse, it is also well-known that it is not uncommon for horses to show a post inhibitory rebound effect, that is, to suddenly panic explosively. The immobility induced by such tactile sensations as twitching may be an adaptive response so that when the horse is in the grip of a predator, it may suddenly and unexpectedly show a burst of energy when the predator, sensing the prey's immobility, momentarily loosens its grip or least expects it.

Tactile stimulation should therefore be used with caution especially when the force applied is high (e.g., during twitching). More knowledge about the tactile sensitivity of horses both during handling and riding is needed to safeguard the welfare of horses and refine our handling techniques. It is likely that horses vary with regard to tactile sensitivity, with individual levels of tactile sensitivity being relatively constant (168). Stereotyping horses is one such group, which have been shown to possess an elevated tactile sensitivity (169). This highlights the need to be extra cautious when applying force to individuals in certain groups of horses. In addition, Saslow (5) suggests in a review (unpublished data) that tactile sensitivity declines with age of the horse and especially so when horses exceed 20 years of age. More knowledge on this topic is generally needed to clarify if such tactile desensitization is caused by aging or habituation. It is believed by many horse people that different breeds of horses differ in their sensitivity. For example what are colloquially known as “cold-blooded” horses are generally deemed less sensitive as they have been selectively bred to endure forces on their bodies from carrying and/or hauling heavy loads, while at the other end of the scale, Arabians and Thoroughbreds are known as “hot-blooded” because of their tactile sensitivity, a catalyst for successful racing. It is also known that prolonged tactile habituation can lead to learned helplessness [where the animal no longer reacts to painful stimuli; (170)] and there is a need for further research into the salience and thresholds of tactile sensitivity in horses. In addition, future research should focus on mapping the tactile sensitivity of the horse's body, and reveal how age, breed, experience and personality may influence the way in which tactile stimulation is perceived by the horse.

## Other Factors Influencing Perception?

### Individuality/Temperament

One common aspect noted in many of the studies included in this review is the large individual variation in sensory abilities and sensitivity. From human research it is known that the sensitivity of different sensory modalities varies from individual to individual with people having different thresholds for noticing, responding to, and becoming irritated with stimuli (171). Similar results have been found in dogs (172), and as these were stable over time, indicating a personality trait, they are used to select guide dogs based in behavioral tests. Personality is defined as a correlated set of individual behavioral and physiological traits that are consistent over time and contexts (173). In horses, personality has been studied, but only sparsely in relation to sensory sensitivity. Mills (174) reviewed individuality and personality in horses and noted that a horse's sensitiveness, (the ease with which performance is affected by environmental disturbance), is important for its welfare, which has also been argued by several other authors. Larose et al. (175) later suggested that the use by the horse of specific eyes to view specific objects or situations (see section Vision), relates to the individual's perception of specific situations, which is further governed by the character of the individual horse. Lansade et al. (168) studied sensory sensitivity in horses with the aim of elucidating whether this could be a stable personality dimension (termed *temperament dimension*). Four stable personality traits, unvarying across context and time, were found: tactile sensitivity, gustato-olfactory sensitivity, auditory sensitivity and visual sensitivity. These results suggest that horses, like humans and other animals, react differently to external stimuli, but with a greater variation between than within individuals. Identifying specific types of horses according to their specific sensory sensitivity could be a way to optimize management and training and may help to improve the welfare of individual horses.

### Season and Circadian Rhythm

A series of studies have looked into seasonality of wild and free ranging domestic horses and found that both Przewalski horses (176, 177) and Shetland ponies (178, 179) are able to adjust their energy budget to accommodate environmental change and predictable changes in forage quality (winter vs. summer quality). This shows that domesticated horses have maintained the capacity for seasonal adaptation to environmental conditions via fluctuations in their metabolic rate. In addition, horses have been found to show an endogenous circadian regulation of muscle function, which show that although horse behavior and activity in general is greatly influenced by external factors including human activities, horses are still influenced endogenously by a natural 24-h internal clock (180). Hence, horse training that follows the natural light conditions might synchronize with the equine circadian rhythm [reviewed in (181)], suggesting that training during dark winter hours should be avoided. Future work could thus focus on determining peak times for training and competing horses in relation to both circadian rhythms and seasonality, to estimate the best training periods and durations throughout the year. It may even be possible to manipulate some aspects of seasonality and circadian rhythms, such as using blue light to stimulate estrus in anestrus mares (182).

## Conclusions

The sensory abilities of horses differ from those of humans in a number of aspects. Equine vision is similar to that of red-green color-blind humans and horses see better in low light than humans. Horses can see almost a full circle around themselves and have a broad rather than a centralized focus They can hear sound frequencies that humans cannot, but unlike most other large land mammals, they hear higher but not lower frequency sounds compared with humans. In addition, horses have a highly developed sense of smell, which is often overlooked, both in equine research as well as training. Horses are very sensitive to touch, but their tactile sensitivity has been very sparsely studied, despite it being used extensively in horse training and handling. The sensory abilities of individual horses may be a stable personality trait, with equine perception affected also by breed, age and in some cases even coat color, highlighting the need to differentiate the care and management of individual horses. There may be unexploited potential of using sensory enrichment/positive sensory stimulation to improve the welfare of horses in various situations e.g., using odors (or signature mixtures), touch or sound to enrich their environment or to appease horses.

Considering the popularity of horses in leisure, sport and other activities, there is a significant need and scope for further research into the sensory abilities of the horse. Knowing how horses perceive their surroundings will help improve awareness of what they find aversive or pleasant and will enable more efficient, welfare-friendly training and handling techniques as well as improve human safety.

## Author Contributions

MR initiated the idea for this review and wrote the first draft. All authors contributed in writing, discussing, proofreading, and fine-tuning the review for publication. All authors contributed to the article and approved the submitted version.

## Conflict of Interest

The authors declare that the research was conducted in the absence of any commercial or financial relationships that could be construed as a potential conflict of interest.
